# Effectiveness of cell adhesive additives in different supramolecular polymers

**DOI:** 10.1002/pol.20210073

**Published:** 2021-03-29

**Authors:** Ronald C. van Gaal, Bastiaan D. Ippel, Sergio Spaans, Muhabbat I. Komil, Patricia Y. W. Dankers

**Affiliations:** ^1^ Laboratory for Cell and Tissue Engineering, Department of Biomedical Engineering Eindhoven University of Technology Eindhoven The Netherlands; ^2^ Institute for Complex Molecular Systems Eindhoven University of Technology Eindhoven The Netherlands; ^3^ Laboratory of Chemical Biology, Department of Biomedical Engineering Eindhoven University of Technology Eindhoven The Netherlands

**Keywords:** bioactive additives, biomaterials, bis‐urea, supramolecular polymers, ureido‐pyrimidinone

## Abstract

Supramolecular motifs in elastomeric biomaterials facilitate the modular incorporation of additives with corresponding motifs. The influence of the elastomeric supramolecular base polymer on the presentation of additives has been sparsely examined, limiting the knowledge of transferability of effective functionalization between polymers. Here it was investigated if the polymer backbone and the additive influence biomaterial modification in two different types of hydrogen bonding supramolecular systems, that is, based on ureido‐pyrimidinone or bis‐urea units. Two different cell‐adhesive additives, that is, catechol or cyclic RGD, were incorporated into different elastomeric polymers, that is, polycaprolactone, priplast or polycarbonate. The additive effectiveness was evaluated with three different cell types. AFM measurements showed modest alterations on nano‐scale assembly in ureido‐pyrimidinone materials modified with additives. On the contrary, additive addition was highly intrusive in bis‐urea materials. Detailed cell adhesive studies revealed additive effectiveness varied between base polymers and the supramolecular platform, with bis‐urea materials more potently affecting cell behavior. This research highlights that additive transposition might not always be as evident. Therefore, additive effectiveness requires re‐evaluation in supramolecular biomaterials when altering the polymer backbone to suit the biomaterial application.

## Graphical abstract

In this study, it is noted that the polymer backbone is of influence on the effective presentation of cell adhesive additives in supramolecular biomaterials. This was observed in both ureido‐pyrimidinone and bis‐urea based supramolecular systems and over different cell types.

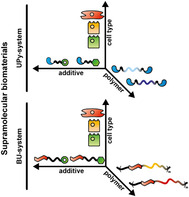

## 1 INTRODUCTION

Tailored biomaterials often rely on covalent modifications or physisorbed coatings to achieve a desired function.^1^, ^2^ Alternatively, biomaterials can be modified by blending functional additives with a base polymer.^3^, ^4^, ^5^ Additive design requires interactions with the base material to ensure stable and effective incorporation. Macromolecular block copolymer additives have previously been employed to introduce anti‐fouling properties in polymers, such as polyurethane though interaction of simple hydrophobic domain.^4^, ^5^, ^6^ More meticulously designed chemical moieties, which can interact specifically through non‐covalent interactions, such as hydrogen bonding and hydrophobic interactions, allow for the generation of complex supramolecular biomaterials.^3^, ^7^ The modular nature provided by the supramolecular domains allows for the construction of highly complex materials with near infinite variation possibilities.^3^ The supramolecular moieties assemble into larger structures to form a biomaterial.^3^, ^7^, ^8^ Conjugation of supramolecular moieties to polymers, small reactive groups, and bioactives allows for the integration of the properties of the separate components as they assemble during processing.^8^, ^9^ Polymers can bring forth variations in degradability and mechanical strength,^10^, ^11^ while reactive groups provide post‐functionalization possibilities,^12^, ^13^ and bioactives can influence cellular interactions^8^, ^9^ for the desired biomaterial. Understanding additive‐polymer interactions is crucial for the design of functional biomaterials.

**FIGURE 1:**
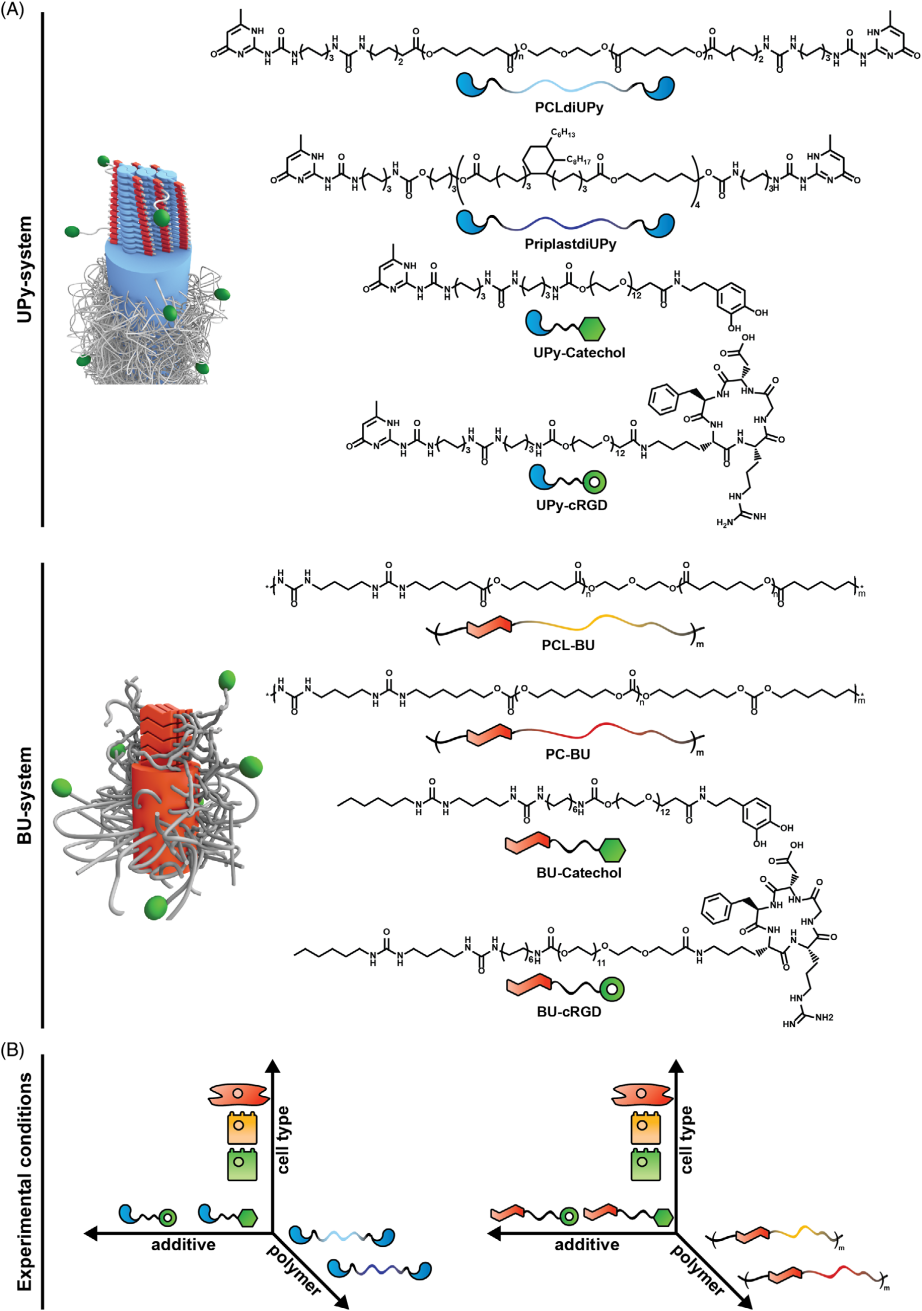
Overview and structures of supramolecular systems. (A) Top panel depicts (UPy)‐system. Left, proposed assembly of UPy‐based fibers, on the right chemical structures of UPy modified polycaprolactone (PCLdiUPy), Priplast (PriplastdiUPy), dopamine (UPy‐catechol), and cyclic RGD (UPy‐cRGD) with schematic representation of the components underneath. Lower panel depicts bis‐urea (BU)‐system. Left, proposed assembly of BU‐based fibers, on the right chemical structures of BU modified PCL (PCL‐BU), polycarbonate (PC‐BU), dopamine (BU‐catechol), and cRGD (UPy‐cRGD) with schematic representation of the components underneath. (B) overview of experimental conditions [Color figure can be viewed at wileyonlinelibrary.com]

Supramolecular materials based on hydrogen bonding ureido‐pyrimidinone (UPy) or bis‐urea (BU) moieties are employed for several biomedical applications.^14^, ^15^ Both supramolecular systems self‐assemble into nano‐fibrous structures that form the hard phase. The assembly mode is different between both systems. UPy‐moieties dimerize through quadruple hydrogen‐bonding, the dimers form stacks through π‐π interactions. The latter are promoted by additional urea or urethane hydrogen bonding separated by a short alkyl spacer from the UPy group (Figure 1(A)).^16^, ^17^, ^18^ It is postulated that three of such stacks form a UPy‐fiber.^19^ BU‐motifs form ribbons through self‐complementary bifurcated hydrogen‐bonding, three to six ribbons assemble laterally into BU‐fibers (Figure 1(A)).^20^, ^21^, ^22^, ^23^

The modular nature of the UPy‐ and BU‐systems allows for effective additives to be incorporated into different supramolecular elastomeric biomaterials.^24^, ^25^ This enables the construction of biomaterial screening libraries.^25^, ^26^ However, the effectiveness of the additive transposition between different supramolecular polymer platforms has been sparsely researched. The nano‐scale self‐assembly of supramolecular materials has been shown to alter with different polymer backbones,^24^, ^27^, ^28^ and additives.^8^, ^29^ Therefore, as both these factors influence assembly, it can be postulated that the same additive is presented differently in the context of different polymer backbones. The non‐trivial transposability of additives in and between supramolecular systems is exemplified by previous research on catechol functionalization. Ceylan et al. functionalized supramolecular peptide amphiphiles with catechols, this improved material adhesion and calcification of the peptide amphiphiles.^30^ Likewise, UPy‐conjugated monomeric catechols (UPy‐Catechol) were shown to improve cell adhesion on poor cell adhesive UPy modified Priplast (PriplastdiUPy).^24^ However, the beneficial cell adhesive properties of UPy‐Catechol were hardly observed when incorporated in UPy modified polycaprolactone (PCL; PCLdiUPy).^24^, ^31^ Remarkably, when BU conjugated catechols (BU‐Catechol) were incorporated in BU modified PCL (PCL‐BU) they enabled long term cell adhesion.^26^ Overall, these reports indicate the importance of understanding the influence of the polymer on additive presentation in different supramolecular systems.

Here, a comprehensive study is presented where cell adhesive additives are transposed between elastomeric supramolecular polymers and supramolecular platforms (Figure 1(A), (B)). This study combines material selections, cell types and experimental read‐outs from previous studies of our group.^24^, ^26^, ^31^, ^36^ The synergistic interactions provide new insights in terms of material behavior and cell specificity, moreover it shows the reproducibility of our previous studies. A small biomaterial library was constructed using two different supramolecular systems (i.e. UPy and BU) while employing two different polymer backbones per system. Within the UPy‐system PCLdiUPy and PriplastdiUPy were selected, and in the BU‐system PCL‐BU and polycarbonate modified with BU motifs (PC‐BU) were chosen as base polymers to investigate the influence of the backbone on additive presentation. Catechol and cyclic RGD (cRGD) were selected as cell adhesive molecules, as they present distinct modes of action which may be differently affected by the polymer systems. Catechol mediated cell adhesion is proposed to be effective through covalent and non‐covalent protein binding by the catechol.^32^, ^33^, ^34^, ^35^ Alternatively, cRGD directly binds to integrins on the cell surface, which enables an intracellular protein recruitment cascade that allows for the formation of focal adhesions, which function as the primary cell adhesive complexes.^1^

Nanostructure assembly after additive incorporation in the different supramolecular polymers was investigated with detailed surface analysis using atomic force microscopy (AFM), water contact angle (WCA), and leakage experiments. Subsequently, three different cell lines were cultured on the biomaterial composites (i.e. Human Kidney 2 cells (HK‐2), renal proximal tubule epithelial cells (RPTEC), and cardiomyocyte progenitor cells (CMPC)) to investigate cell specific responses to the biomaterial interface. The cell lines were selected in the context of previous research conducted in our group. HK‐2 focal adhesion morphology allowed for the investigation of cRGD levels on UPy‐ and BU‐based polymer films.^36^ RPTECs were employed to screen for long‐term beneficial effects of BU‐peptides and BU‐Catechol.^26^ Finally, CMPCs adhesion indicated the effectiveness of UPy‐Catechol to promote cell adhesion in different UPy‐polymers.^24^ In this study, cell adhesion, cell spreading and focal adhesion properties were quantified to assess cell response to the materials.

## 2 EXPERIMENTAL

### 2.1 Polymer film preparation

UPy‐Catechol, UPy‐cRGD, BU‐Catechol and BU‐cRGD were synthesized as reported previously.^26^, ^31^, ^36^ PCLdiUPy (*M*_n_ = 2.8 kg/mol), PriplastdiUPy (*M*_n_ = 5.0 kg/mol) PCL‐BU (*M*_n_ = 2.7 kg/mol per segment), PC‐BU (*M*_n_ = 2.4 kg/mol per segment) were acquired from SyMO‐Chem (The Netherlands). To prepare functionalized polymer films, PCLdiUPy, PCL‐BU and PC‐BU were dissolved in hexafluoroisopropanol (20 mg/ml; HFIP, Fluorochem) and 5 mol% of corresponding UPy‐Catechol, UPy‐cRGD, BU‐Catechol, or BU‐cRGD was added. Pristine films were produced from the functionalized polymer solutions. To yield a clear polymer films, a 45 μl polymer solution droplet was cast on 14 mm Ø glass coverslips and left to evaporate at a maximum relative humidity of 40%. Films were kept at least overnight to ensure HFIP evaporation. The hydrophobic nature of PriplastdiUPy required specialized casting conditions to produce polymer films on glass coverslips, the method has been previously reported by Spaans et al.^24^ In short, a 40 mg/ml PriplastdiUPy solution was prepared in chloroform. UPy‐Catechol and UPy‐cRGD were dissolved chloroform: methanol (95:5) in to create 8.3 mM additive solutions. The solutions were mixed to yield no or 5 mol% functionalization of the additive in the polymer solution. PriplastdiUPy solutions were casted 80 μl on silanized glass coverslips and left to evaporate at a maximum relative humidity of 40%. Films were kept at least overnight to ensure chloroform evaporation.

### 2.2 Surface characterization

A Digital Instruments Multimode Nanoscope IIIa, operating in the tapping mode regime, was used to record phase and height images of solution‐cast films at room temperature with silicon cantilever tips (PPP‐NCHR, NanoSensors^tm^, 204–497 kHz, 10–130 N/m). Images were processed using Gwyddion software (version 2.43). Polymer film hydrophobicity was determined by water contact angle analysis. A 4 μl water droplet was deposited on the polymer surface, images were taken at 5 s after droplet deposition and contact angles were measured with contact angle system OCA and SCA 202 v4.1.13 build 1020 software (Dataphysics Intruments, Germany). Two droplets per sample were deposited over 3 replicates.

### 2.3 Additive leakage measurements

Polymer films were incubated with 500 μl PBS for 24 h at RT. Potential leakage of the UPy‐ or BU‐additives in the supernatant was analyzed by using LC–MS measurements, with a water‐acetonitrile mobile phase enriched with 0.1% v/v formic acid. All experiments were performed in triplicate. The standard curve was prepared by dissolving the additives in a mixture of Milli‐Q water and/or acetonitrile: UPy‐Catechol (Milli‐Q water:Acetonitrile 3:1 [v/v]), UPy‐cRGD (Milli‐Q water), BU‐Catechol (Milli‐Q water:Acetonitrile 1:1 [v/v]) and BU‐cRGD (Milli‐Q). The UPy‐catechol solutions were diluted to 40, 10, 2.5, 0.3, and 0.2 μg/ml. The UPy‐cRGD solutions were diluted to 57, 14.3, 3.6, 0.5, and 0.2 μg/ml. The BU‐Catechol solutions were diluted to 28, 7, 1.8, 0.9, and 0.2 μg/ml. The BU‐cRGD solutions were diluted to 56, 14, 3.5, 0.4, and 0.2 μg/ml. The total ion count was determined by the genesis algorithm. Samples were kept at −20°C during storage and at 4°C during analysis.

### 2.4 Cell culture

Human kidney 2 cells (HK‐2; ATCC, Germany) were cultured in Dulbecco's Modified Eagle Medium (DMEM; 41966, Gibco, UK), supplemented with 10% v/v fetal bovine serum (FBS; Greiner Bio‐one, The Netherlands) and 1% v/v penicillin and streptomycin (penstrep, Invitrogen). CMPCs (Leiden University Medical Center, The Netherlands) were immortalized by lentiviral transduction of hTert and BMI‐1.^37^ Culture medium consisted of SP++ growth medium containing M199 (Gibco) supplemented with 33% v/v EGM‐2 BullitKit (Lonza), 10% v/v FBS, 1% v/v penstrep, 1% v/v non‐essential amino acids (Gibco). RPTEC cells (RPTECs‐TERT1; ATCC) were cultured in complete medium consisting of DMEM:F‐12 Nutrient Mixture (Gibco) containing L‐Glutamine and 15 mM 4‐(2‐hydroxyethyl)‐1‐piperazineethanesulfonic acid. Furthermore, the medium was supplemented with 1% v/v penstrep, RPTEC growth kit (ATCC, PCS‐999‐058, PCS‐999‐059) and 0.1 mg/ml G418 (Sigma‐Aldrich). All three cell types were cultured under a humidified atmosphere at 37°C and 5% CO_2_. Polymer films were sterilized with UV irradiation for 10 min and subsequently mounted in custom inserts to prevent film detachment during culture. The seeding densities for 1 day culture were 10*10^3^ cells/cm^2^ (HK‐2), 26*10^3^ cells/cm^2^ (CMPC), and 30*10^3^ cells/cm^2^ (RPTEC). Long‐term monolayer formation of RPTEC was assessed after a 3 week culture period and started with an initial seeding density of 1.6*10^5^ cells/cm^2^. Medium was changed every 2 to 3 days. Three replicates were performed for each experiment.

### 2.5 Immunofluorescent staining

Cells were fixated at their respective time points. CMPC and RPTEC were exposed to 3.7% v/v formaldehyde (Merck) in PBS (Sigma‐Aldrich) for 10 min and permeabilized with 0.5% v/v Triton X‐100 (Merck) in PBS for 10 min. HK‐2 s were fixated and permeabilized simultaneously with 3.7% v/v formaldehyde 0.5% v/v Triton X‐100 in PBS for 10 min. All afore mentioned steps were preceded and succeeded by PBS washing steps. HK‐2 and RPTEC samples were blocked with 5% w/v BSA (Roche) for 20 min at RT. Short term cultured HK‐2 and RPTEC samples were incubated with mouse‐α‐pFAK antibody (1:400 dilution, BD Bioscience, 611,723), and long term RPTEC samples were incubated with mouse‐α‐ZO‐1 antibody (1:200 dilution, BD Biosciences, 610,966) in staining buffer (2% w/v BSA, 0.05% v/v Triton X‐100 in PBS) for 60 min at RT. Samples were washed with 0.05% v/v Triton X‐100 in PBS, and subsequently incubated with Goat‐α‐Mouse‐Alexa Fluor 555 (1:200, Molecular Probes, A21424) and phalloidin‐Atto 488 (1:300, Sigma‐Aldrich) in staining buffer for 45 min with 4′‐6‐diamidino‐2‐phenylindole (DAPI; 0.1 μg/ml; Sigma‐Aldrich) added in the last 10 min. CMPC samples were blocked for non‐specific antibody binding with 2% v/v horse serum (Gibco) in PBS for 20 min. Subsequently, samples were incubated with rabbit‐α‐zyxin antibody (1:400, Sigma‐Aldrich, HPA004835) in 10% horse serum in PBS for 2 h at 4°C. Cells were washed with PBS and incubated with Goat‐α‐Rabbit‐Alexa Fluor 555 (1:300, Molecular Probes, A21428) and phalloidin‐Atto 488 for 1 h in PBS at RT. The secondary antibody was aspirated and cells were stained with DAPI for 5 min in PBS. HK‐2, CMPC and RPTEC samples were washed with PBS and mounted with mowiol for fluorescent imaging. Fluorescent images were acquired with a Zeiss Axiovert 200 M microscope and AxioVision software (Zeiss, Germany).

### 2.6 Fluorescence image analysis

Cell area and FA size and number per cell were quantified with a custom‐built Mathematica analysis script (version 11.1, Wolfram Research Inc.), which has been previously reported in literature.^36^, ^38^ Analysis was performed on 5 to 12 single cells per sample in each of the three replicates. Focal adhesions within the size range of 0.1 and 11 μm^2^ were used for further analysis. Cell–cell contacts were quantified through previously developed custom Matlab script (version R2015a, The MathWorks Inc.).^26^ In short, a 20 by 20 line grid is superimposed on the image. Intersections between the grid and the ZO‐1 staining are quantified.

### 2.7 Statistics

Data with n ≤ 5 were subjected to a Kruskal‐Wallis test with a Dunns post‐test, while data with n > 5 were subjected to a one way ANOVA followed by a Bonferroni post‐test or a student *t* test. Experimental conditions were compared to pristine samples with the same backbone polymer in post‐tests. For data regarding focal adhesions, post‐test were compared between all conditions. Tests were performed with the use of Prism 5 (GraphPad Software Inc.). Probabilities of *p* ≤ 0.05 were considered as significantly different.

## 3 RESULTS AND DISCUSSION

### 3.1 Backbone polymer and additive influence surface properties

Taken together, the results indicated that both the backbone polymer and the supramolecular additive influence the assembly of hard phase fibers and stable additive incorporation in both supramolecular systems. The addition of additives in the UPy‐system mainly resulted in alterations of the nano‐fiber length. Moreover additives in PCLdiUPy induced the formation of random aggregates on the surface. BU‐additives elicited profound changes in surface morphology after addition, either platelet‐like or fibrous aggregates presented on the surface. Curiously, the inclusion of BU‐additives appeared to promote local alignment and growth of hard‐phase fiber in PC‐BU, yet not in PCL‐BU.

**FIGURE 2:**
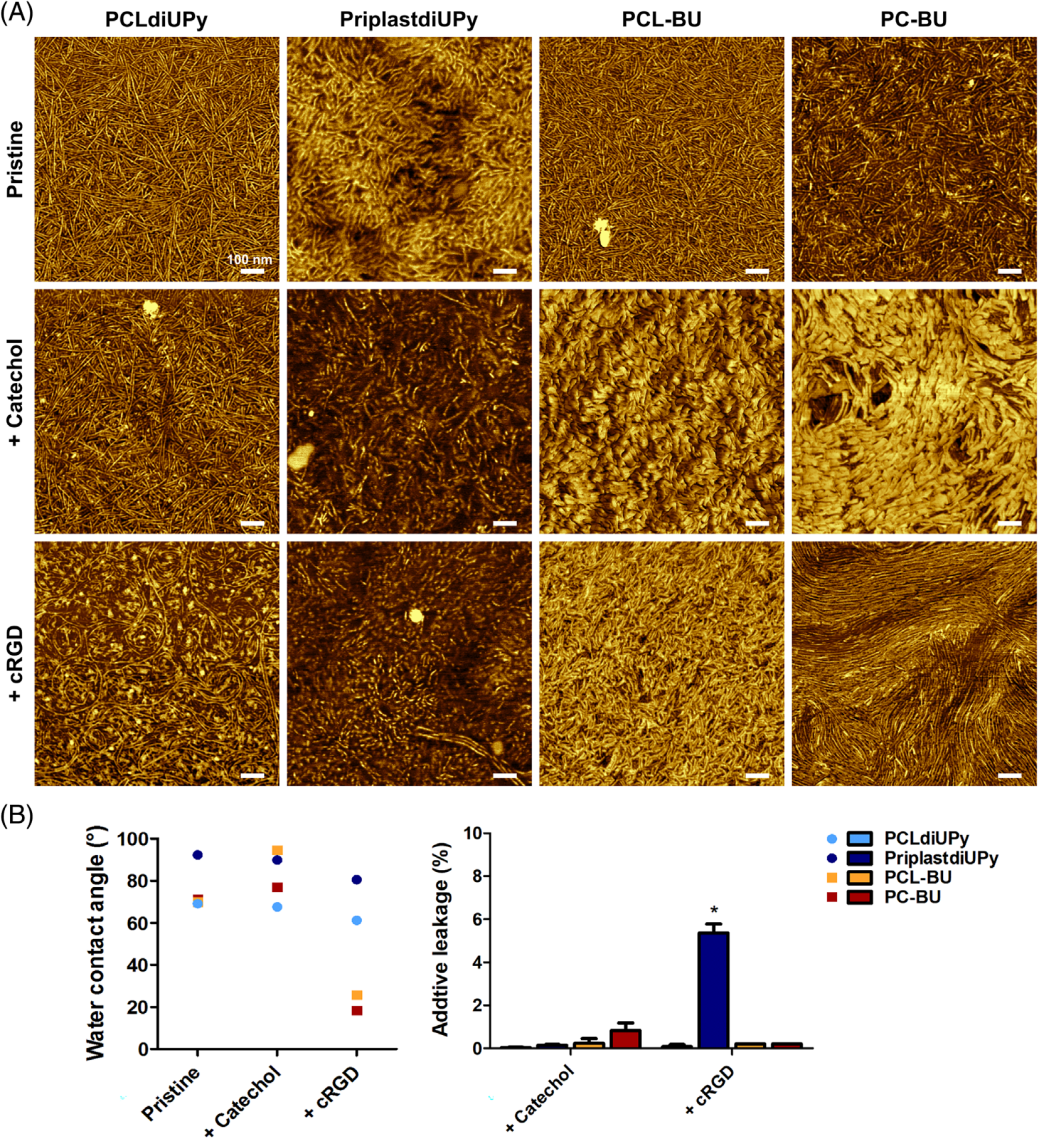
Influence of backbone polymer and additive on surface morphology. (A) AFM phase micrographs of solution cast films of polymer and additive combinations. Selected polymers from left to right, PCLdiUPy, PriplastdiUPy, PCL‐BU, and PC‐BU. Additive functionalization from top to bottom, no additive (pristine), 5 mol% UPy‐catechol or BU‐catechol applied in corresponding supramolecular base polymer (catechol), and 5 mol% UPy‐cRGD or BU‐cRGD applied in corresponding supramolecular base polymer (cRGD). Scale bars are 100 nm. (B) Water contact angle measurements (left) and additive leakage measurements (right) of afore mentioned polymer and additive combinations. Data of three replicates, mean ± standard error of the mean. *p ≤ 0.05, intra backbone comparison [Color figure can be viewed at wileyonlinelibrary.com]

All pristine supramolecular polymers exhibited hard phase fiber formation; however, all have a unique morphology (Figure 2(A)).^22^, ^24^ The nanoscale fibers observed in PCLdiUPy were long and appear orientated in random directions. PriplastdiUPy films presented shorter hard phase fibers compared to PCLdiUPy (Figure 2(A)). PriplastdiUPy surfaces showed a three times larger height difference than PCLdiUPy (Figure S1). To assess the hydrophilic character of the polymer surfaces water contact angles were determined, PCLdiUPy presented an angle of 69.1 ± 0.4°, while pristine PriplastdiUPy was drastically more hydrophobic with a WCA of 92.3 ± 1.1° (Figure 2(B)). Pristine PCL‐BU exhibited nanofiber morphology resembling that of PCLdiUPy (Figure 2(A)). PC‐BU nanoscale fibers appear to consist out of a mixed population, consisting out of longer subset and a shorter subset (Figure 2(A)). The hydrophilicity of PCL‐BU and PC‐BU did not significantly differ between each other, or with PCLdiUPy (Figure 2(B)). The observed alterations in UPy‐fiber size possibly originate from two differences between the PCLdiUPy and PriplastdiUPy. Firstly, the stacking of UPy‐dimers in the longitudinal direction is aided by hydrogen bonding between urea (PCLdiUPy) or urethane (PriplastdiUPy) motifs (Figure 1(A)).^19^, ^28^ Urea motifs have the capacity to form two hydrogen bonds, while the urethane motif forms a sole hydrogen bond, which is less capable to stabilize fiber growth.^18^ Secondly, Priplast is an semi‐crystalline polymer due its hydrophobic bulky backbone, containing cyclohexane with alkane side chains, which has been shown to hinder crystallization in copolymers with polylactide.^39^ Slight differences in nanofiber morphology between PCL‐BU and PC‐BU likely originate from the backbone, as the BU motif is equal in both polymers, unlike in the UPy‐system were a urea or urethane is present. The backbone has also been shown to affect the melt energy required for BU‐stacks.^40^

#### 3.1.1 Surface characteristics of UPy‐additive functionalized materials

The addition of UPy‐Catechol to PCLdiUPy resulted in appearance of disperse punctuated aggregates on the biomaterial surface (Figure 2(A)). The morphology of the nanofibers appeared not to be affected for PCLdiUPy. The incorporation of UPy‐cRGD into PCLdiUPy yielded globular aggregates at the material surface, more frequent and larger than observed after UPy‐Catechol addition to PCLdiUPy (Figure 2(A)). Moreover, a fraction of the nano‐scale fibers appeared to increase in length. The surface height difference was not affected by additive addition compared to pristine PCLdiUPy (Figure S1). Both functionalization of PCLdiUPy with UPy‐Catechol or UPy‐cRGD led to slight non‐significant reduction of surface hydrophobicity compared to pristine PCLdiUPy (Figure 2(B)). For both additives retention was high, 100 ± 0.03% UPy‐Catechol and 99.9 ± 0.1% UPy‐cRGD was retained (Figure 2(B)). The addition of UPy‐Catechol or UPy‐cRGD to PriplastdiUPy did not induce clear aggregate formation on the surface when compared to pristine PriplastdiUPy (Figure 2(A)). Nano‐scale fibers appeared to decrease in length after introduction of UPy‐Catechol or UPy‐cRGD to PriplastdiUPy, in contrast to the effects observed in PCLdiUPy. The surface height difference diminished in PriplastdiUPy films with additives compared to pristine (Figure S1). WCAs were non‐signifcantly reduced after UPy‐Catechol and UPy‐cRGD incorporation in PriplastdiUPy (Figure 2(B)). UPy‐Catechol was effectively retained in PriplatdiUPy, 99.9 ± 0.03%; however, significant UPy‐cRGD leakage was observed 5.4 ± 0.42% (Figure 2(B)). The reduction in PriplastdiUPy hard phase fibers after UPy‐additive addition is speculated to be the result of a mismatch between the PriplastdiUPy's urethane and the UPy‐additives' urea‐group (Figure 1(A)). Interestingly, PCLdiUPy presented surface aggregations, which have been postulated to be the UPy‐cRGD exposed at the surface,^36^ while in PriplastdiUPy none were observed. This might indicate similar levels of peptide exposed on the surface, but in different patterns. However, leakage experiments indicated that UPy‐cRGD is not stably incorporated in PriplastdiUPy.

#### 3.1.2 Surface characteristics of BU‐additive functionalized materials

Functionalization of PCL‐BU with BU‐Catechol caused the appearance of platelet‐like structures at the surface (Figure 2(A)). The surface height difference increased at least 3 times after the BU‐Catechol incorporation in PCL‐BU (Figure S1). Moreover, a strong increase in hydrophobicity was observed after BU‐Catechol functionalization of PCL‐BU compared to pristine, yielding a WCA of 94.7 ± 0.2° (Figure 2(B)). The assembly of BU‐cRGD with PCL‐BU resulted in complete coverage with fibril aggregates on the biomaterial surface (Figure 2(A)). Coincidingly, a drastic increase in hydrophilicity was observed compared to pristine PCL‐BU (Figure 2(B)). Both BU‐Catechol and BU‐cRGD were effectively retained in PCL‐BU, leakage was determined to be 0.24 ± 0.22% and ≤ 0.2%, respectively (Figure 2(B)). Platelet‐like structures, that increased the surface height difference, were also observed on PC‐BU surfaces after BU‐Catechol incorporation (Figure 2(A), S1). The BU‐Catechol structures on the PC‐BU surface appeared to be more aligned compared to those presented on the PCL‐BU surface. The addition of BU‐Catechol to PC‐BU yielded an increase in surface hydrophobicity compared to pristine (Figure 2(B)). The addition of BU‐cRGD to PC‐BU did not result in clear aggregate formation. However, BU‐cRGD did induce an elongation and local alignment of the BU‐fibers compared to pristine PC‐BU (Figure 2(A)). PC‐BU with BU‐cRGD resulted in a strong decrease of surface hydrophobicity compared to pristine BU‐materials (Figure 2(B)). BU‐Catechol showed low amounts of leakage from the material, 0.83 ± 0.35% additive leakage, BU‐cRGD showed stable incorporation of ≤0.2% additive leakage (Figure 2(B)).

Catechol functionalization yielded vastly different surface morphologies in the UPy‐system compared to BU functionalized materials (Figure 2(A)). The platelets are assumed to be crystalline OEG spacer domains on the surface based morphological similarity (Figure 1(A)).^25^ Catechols can be both hydrophilic and hydrophobic in nature depending on the configuration, here most likely the hydrophobic properties dominate over the hydrophilic OEG spacers.^25^, ^32^, ^33^, ^41^ The platelets were stably incorporated, with low levels of leakage. The random aggregate formation on PCL‐BU surfaces after BU‐cRGD incorporation is consistent with previous results.^36^ In contrast are the elongated nano‐fibers on PC‐BU surfaces, which present local alignment thereby creating a “Van Gogh style”‐effect. This behavior can also be faintly observed after the addition of BU‐Catechol to PC‐BU. The PC melt is lower compared to PCL, indicating that PC has less crystallization potential at room temperature.^40^ Fiber assembly in PC‐BU is therefore dominated by BU‐assembly, with little polymer crystallization interference, additives likely promote fiber elongation. The hydrophilic nature of the OEG‐spacer and amino acids in the peptide of the BU‐additive can be attributed to the increase in hydrophilicity after BU‐cRGD functionalization in PCL‐BU and PC‐BU (Figure 1(A)). Previous results revealed the same trend that BU‐based materials more readily increase hydrophilicity after cRGD functionalization than UPy‐based materials.^36^ Moreover this increase was positively correlated to the level of functional cRGD at the surface.

### 3.2 Cell specific adhesion as function of biomaterial composition

The cell adhesive properties of the supramolecular biomaterials, and the adhesive monomeric catechols and cRGD functionalizations have often been explored with the use of one cell type in one supramolecular system, both the additive or cell type differed per study.^24^, ^31^ Cell specificity has however been shown to influence the level of adhesion to biomaterials.^42^ Three different cell types were seeded on the different biomaterial compositions to assess the effect of the polymer backbone and adhesive molecule on possible cell specific adhesion.

In summary, similar trends in cell adhesion were observed towards the small library of biomaterials, although minor cell specific and medium induced differences were present. Pristine PCLdiUPy, PCL‐BU and PC‐BU were found to be cell adhesive, while PriplastdiUPy reduced cell spreading and long‐term adhesion. UPy‐Catechol addition was overall effective in improving cell adhesion on PriplastdiUPy, while no effect was observed in PCLdiUPy. The addition of UPy‐cRGD showed opposite effect, no improved cell adhesion in PriplastdiUPy, improved cell adhesion on PCLdiUPy. This indicated that the polymer backbone can influence the effectiveness of a cell adhesive additive. The addition of BU‐Catechol to PCL‐BU and PC‐BU was found to be detrimental for cell adhesion when media was supplemented with serum. BU‐cRGD addition resulted in overall improved cell adhesion on modified PCL‐BU, but when incorporated in PC‐BU BU‐cRGD detrimental effects on cell adhesion.

**FIGURE 3:**
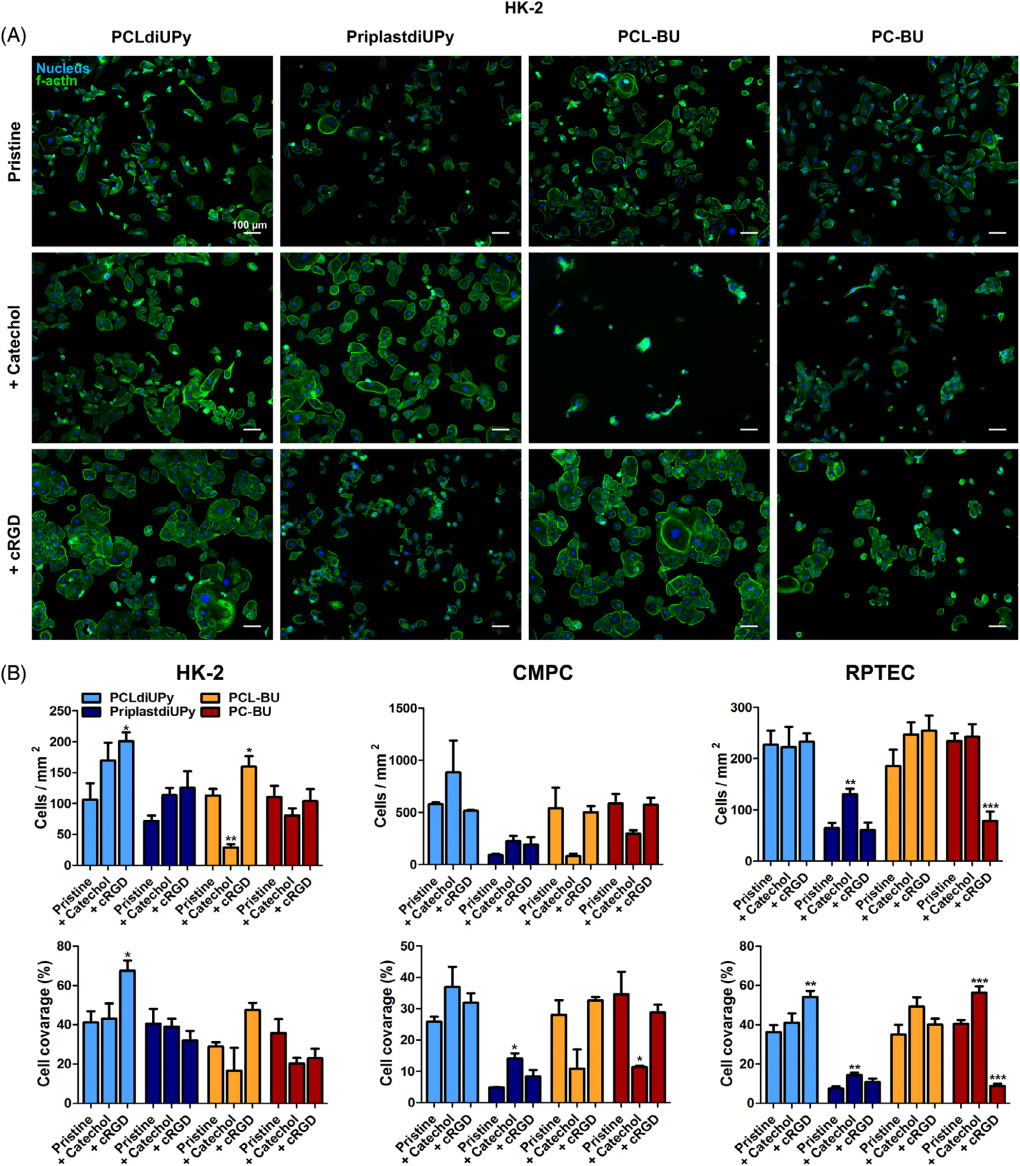
Cell adhesion to supramolecular materials. (A) HK‐2 cells cultured for 24 h on biomaterials, stained for f‐actin (green) and the nucleus (blue), scale bars are 100 μm. Selected polymers from left to right, PCLdiUPy, PriplastdiUPy, PCL‐BU, and PC‐BU. Additive functionalization from top to bottom, no additive (pristine), 5 mol% UPy‐catechol or BU‐catechol applied in corresponding supramolecular base polymer (catechol), and 5 mol% UPy‐cRGD or BU‐cRGD applied in corresponding supramolecular base polymer (cRGD). (B) quantification of cells/mm^2^ (top row) and cell coverage (bottom row) over the biomaterial surface from fluorescent images of HK‐2 (left), CMPC (middle), and RPTEC (right). Data of three replicates, mean ± standard error of the mean. *p ≤ 0.05, **p ≤ 0.01, and ***p ≤ 0.001 intra backbone compared to pristine [Color figure can be viewed at wileyonlinelibrary.com]

Pristine PCLdiUPy allowed for cell adhesion and spreading of all three different cell types (Figure 3, S2‐3). PriplastdiUPy showed a reduction in adhesive cells and cell spreading for HK‐2, CMPC and RPTEC compared to pristine PCLdiUPy. The spreading of HK‐2 cells was not as strongly affected as spreading of CMPCs and RPTECs on PriplastdiUPy (Figure 3(B)). Long‐term culture of RPTECs on pristine PriplastdiUPy revealed cell detachment after initial adhesion (Figure S3). Pristine PCL‐BU and PC‐BU elicited a similar cell response by all three cell types as observed for pristine PCLdiUPy, (Figure 3, S2‐3). These results are unsurprising as pristine PCLdiUPy, PCL‐BU, and PC‐BU have all been shown to be adherent for multiple cell types in literature.^15^, ^24^, ^36^, ^43^ The poor cell adhesive properties of PriplastdiUPy displayed the same trend with previous results.^24^ The PriplastdiUPy surface was shown to be hydrophobic (Figure 2(B)), such surfaces tend to denature absorbed proteins, which might prevent the adhesion of cells to those proteins.^44^

#### 3.2.1 Cell adhesion on UPy‐additive functionalized materials

The addition of UPy‐Catechol to PCLdiUPy did not influence cell adhesion and spreading for all cell types compared to pristine PCLdiUPy (Figure 3, S2‐3). The addition of UPy‐cRGD to PCLdiUPy increased the number of adhered cells and cell spreading for HK‐2 cells compared to pristine PCLdiUPy. Cell numbers for CMPCs and RPTECs were comparable after UPy‐cRGD functionalization to pristine PCLdiUPy (Figure 3, S2‐3). However, for RPTECs spreading was improved on cRGD functionalized PCLdiUPy. PriplastdiUPy functionalized with UPy‐Catechol saw a trend of increased cell adhesion and spreading for all three cell types compared to pristine PriplastdiUPy (Figure 3(B)). Long‐term culture of RPTECs revealed larger cell clusters on UPy‐Catechol functionalized PriplastdiUPy compared to 24 h culture (Figure S3). PriplastdiUPy functionalized with UPy‐cRGD showed an improved trend in the number of HK‐2 s and CMPCs adhering to the material compared to pristine PriplastdiUPy, but spreading was not improved (Figure 3, S2A). RPTECs showed comparable poor cell adhesion and spreading between pristine and UPy‐cRGD functionalized PriplastdiUPy (Figure 3(B), S2B). RPTECs detached from UPy‐cRGD modified PriplastdiUPy surfaces over time (Figure S3).

The ineffectiveness of UPy‐Catechol to improve adhesion and spreading on PCLdiUPy is comparable to previous results performed with CMPCs and a different renal epithelial cell line.^24^, ^31^ The influence of the backbone polymer on the functionality of UPy‐Catechol was noted before.^24^, ^31^ It has been speculated that the innate cell adhesive character of PCLdiUPy might dominate over the cell adhesive effects brought by monomeric catechols.^31^ PriplastdiUPy is poor cell adhesive and the adhesive effect of catechols prevails. Additionally, the level of UPy‐Catechol presentation at the surface might be affected by the polymer backbone. However, to our knowledge there is no method available to quantify surface catechols within our supramolecular systems. The improved cell spreading on PCLdiUPy surfaces after UPy‐cRGD incorporation is a sign of effective cRGD at the surface.^1^ UPy‐cRGD did not improve cell adhesion in PriplastdiUPy, and cell detachment was observed over longer culture periods. This is mostly likely caused by the leakage of UPy‐cRGD from PriplastdiUPy (Figure 2(B)), soluble RGD is known effector for cell detachment.^1^ These results demonstrate that not every cell adhesive additive is effective in a different polymer system.

#### 3.2.2 Cell adhesion on BU‐additive functionalized materials

Introduction of BU‐Catechol in PCL‐BU and PC‐BU had a detrimental effect on cell adhesion of HK‐2 s and CMPCs compared to their pristine counterparts, and the effect was more prominent in functionalized PCL‐BU (Figure 3, S2A). On the other hand, RPTEC remained unaffected by BU‐Catechol functionalization (Figure 3(B), S2B). Only BU‐Catechol mixed with PCL‐BU and in PC‐BU showed successful functionalization to maintain RPTEC monolayers with near perfect coverage over a prolonged culture period of 3 weeks (Figure S3). Media composition has been shown to affect cell material interactions and might explain the observed differences between HK‐2 and CMPC, and RPTEC.^45^ RPTEC culture media was not supplemented with serum, while that of HK‐2 and CMPC did contain it. The introduction of serum in culture media reduced cell adhesion of RPTEC on BU‐Catechol functionalized PCL‐BU and PC‐BU surfaces compared to pristine PCL‐BU and PC‐BU (Figure S4). PCL‐BU functionalization with BU‐cRGD resulted in improved cell adhesion and spreading of HK‐2 s compared to pristine PCL‐BU (Figure 3). CMPCs and RPTECs showed comparable adhesion and spreading between pristine and functionalized PCL‐BU with BU‐cRGD (Figure S2‐3). The introduction of BU‐cRGD to PC‐BU resulted in comparable cell adhesion for HK‐2 and CMPC to pristine PC‐BU (Figure 3(A), S2A). HK‐2 s might reduce spreading, while CMPC showed similar spreading after BU‐cRGD functionalization of PC‐BU (Figure 3(B)). RPTECs showed a drastic decrease in cell amount and spreading after BU‐cRGD functionalization of PC‐BU (Figure 3(B), S2B). However, long‐term culture of RPTECs resulted in equally confluent monolayers in BU‐cRGD modified PCL‐BU and PC‐BU, both outperforming monolayers on pristine materials (Figure S3).

It remains to be elucidated why the presence of serum deters cell adhesion on BU‐catechol modified polymers. The catechol rich surfaces might bind proteins in an unfavorable conformation, which can decrease cell adhesion. Furthermore the addition of BU‐cRGD to PCL‐BU and PC‐BU resulted in strikingly different results for cell adhesion. This is potentially caused by the different surface morphologies of the materials, as both materials showed stable cRGD incorporation. The distance and pattern of RGD presentation has been shown to affect its effectiveness.^46^, ^47^ Therefore, BU‐cRGD might be presented in a less favorable pattern in the longer PC‐BU nano‐fibers reducing the cell adhesion compared to PCL‐BU surfaces. Alternatively, the level of available BU‐cRGD on the surface might be lower on PC‐BU compared to PCL‐BU.

### 3.3 Focal adhesion behavior on biomaterial compositions

**FIGURE 4:**
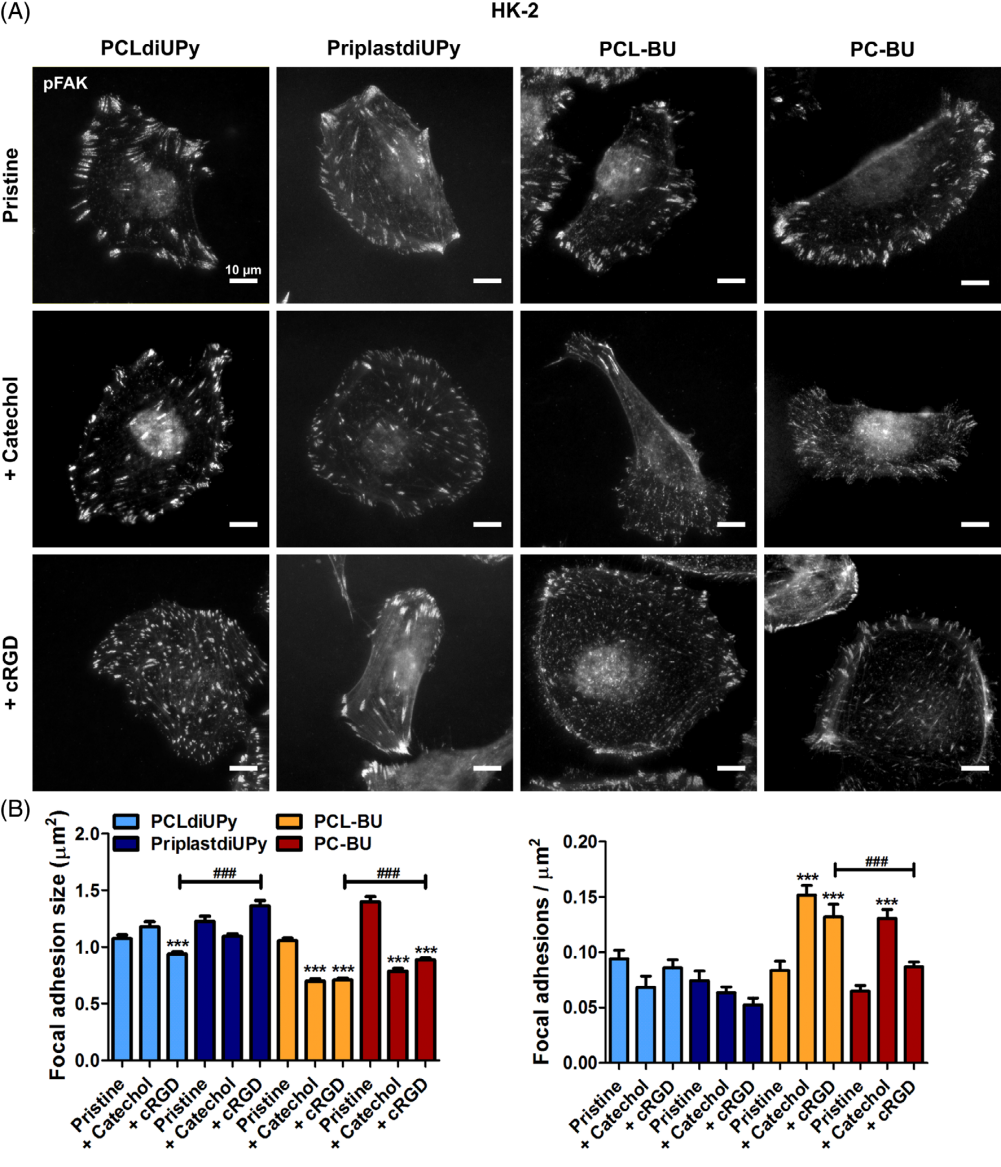
Focal adhesion morphological properties on supramolecular biomaterials. Fluorescence microscopy images of HK‐2 cells cultured for 24 h on PCLdiUPy (left column), PriplastdiUPy (center left column), PCL‐BU (center right), or PC‐BU (right column) films. Additive functionalization from top to bottom, no additive (pristine), 5 mol% UPy‐catechol or BU‐catechol applied in corresponding supramolecular base polymer (catechol), and 5 mol% UPy‐cRGD or BU‐cRGD applied in corresponding supramolecular base polymer (cRGD). Cells stained for pFAK, scale bars are 10 μm, *n = 3*. Bottom graphs depict analysis of pFAK containing focal adhesions to assess size and focal adhesions/cell area. ***p ≤ 0.001, intra backbone compared to pristine; ###*p* ≤ 0.001, inter backbone in the same supramolecular systems, mean ± standard error of the mean depicted [Color figure can be viewed at wileyonlinelibrary.com]

The focal adhesions were investigated to acquire more in depth knowledge on the adhered potential of the cells on various biomaterial compositions. Focal adhesions modulate their properties in response to the functional cRGD level at the surface, they become more plentiful and reduce in size as the cRGD concentration is increased.^48^ This property can be exploited to get a semi‐quantitative measurement of functional cRGD at the surface when comparing the different biomaterials. The employed CMPCs, and RPTEC presented minute focal adhesions in great number, which we were unable to quantify with conventional image analysis software (Figure S5). Moreover to exclude potential effects on focal adhesion behavior through stress distribution over cell–cell contacts, single cells are preferred for analysis.^49^, ^50^ Overall no definitive conclusions could be drawn for qualitative analysis of the CMPC and RPTEC images (Figure S5). Therefore analyses only focuses on the focal adhesions presented by the HK‐2 cells (Figure 4).

Together these results showed that focal adhesion behavior to additives in the UPy‐based polymers was less pronounced than in the BU‐system. The polymer backbone in both systems influenced the effectiveness of cRGD presentation. The origin of the contrast found between functional cRGD levels in PCL‐BU and PC‐BU remains to be elucidated. Whether it depends on the surface concentration, the mode of presentation within the supramolecular fibers, or fiber orientation.

All pristine materials induced mature focal adhesions with sizes between 1.06 ± 0.02 to 1.39 ± 0.05 μm^2^, in descending order of induced size PC‐BU, PriplastdiUPy, PCLdiUPy to PCL‐BU (Figure 4). No significant differences were found in the amount of focal adhesions per cell area, although a similar trend was observed as with focal adhesion size, in ascending order of FAs per cell area PC‐BU, PriplastdiUPy, PCL‐BU to PCLdiUPy (Figure 4).

#### 3.3.1 Focal adhesion behavior on UPy‐based materials

No significant differences were observed in focal adhesion properties on UPy‐Catechol functionalized PCLdiUPy or PriplastdiUPy compared to their pristine counterparts (Figure 4). Incorporation of UPy‐cRGD in PCLdiUPy significantly reduced focal adhesion size, but not focal adhesion density compared to pristine PCLdiUPy (Figure 4). No significant differences were observed in focal adhesion properties for PriplastdiUPy compared to pristine PriplastdiUPy (Figure 4). PriplastdiUPy with UPy‐cRGD had significantly larger focal adhesions compared PCLdiUPy with UPy‐cRGD. Although no differences were observed in focal adhesions between pristine PriplastdiUPy and PriplastdiUPy functionalized with UPy‐Catechol conditions, the latter did improve the number of adhered cells. Additionally, long‐term culture of RPTEC showed cell detachment if UPy‐Catechol was not present in PriplastdiUPy, indicating that focal adhesion properties might not be indicative for long‐term cell adhesive responses. HK‐2 s presented smaller and more focal adhesions on cRGD modified PCLdiUPy than on pristine PCLdiUPy and on modified PriplastdiUPy, this indicated that more functional cRGD is presented on PCLdiUPy surfaces than on PriplastdiUPy surfaces, in line with reported cRGD leakage (Figure 2(B)).^48^

#### 3.3.2 Focal adhesion behavior on BU‐based materials

BU‐Catechol functionalization significantly decreased focal adhesion size and increased focal adhesions per cell area in both PCL‐BU and PC‐BU compared to pristine (Figure 4). Note that BU‐Catechol functionalization reduced cell adhesion (Figure 3), which likely induced small and plentiful focal adhesion by a different mechanism. Incorporation of BU‐cRGD in PCL‐BU resulted in a significant reduction focal adhesion size and increase in focal adhesion density compared to pristine PCL‐BU (Figure 4). PC‐BU functionalized with BU‐cRGD induced a significant decrease in focal adhesion size, but no increase was observed in focal adhesion density compared to pristine PC‐BU (Figure 4). Both focal adhesion size and density significantly differed between BU‐cRGD functionalized PCL‐BU and PC‐BU. The negative correlation between functional cRGD at the surface and focal adhesion size indicates that PCL‐BU presents more functional cRGD at the surface when compared to PC‐BU.^48^ Moreover, the same reasoning indicates that the BU‐system better presents cRGD than the UPy‐system.

This research sheds light on the influence of the supramolecular additive and the polymer backbone on surface assembly of UPy‐ and BU‐fibers. Over the years supramolecular additive incorporation in polymers has been extensively studied; however, often both the polymer backbone, additive design, and additive end‐moiety were varied.^25^, ^29^, ^51^, ^52^ The effect of additive and backbone polymer could therefore for a long time not be decoupled to assess the influence nano‐scale assembly in supramolecular elastomeric polymer systems. The end group of the supramolecular additive was revealed to greatly affect the surface morphology in both the UPy and BU‐system in recently published papers.^25^, ^36^, ^52^ Previous research hinted toward the importance of the polymer backbone for nano‐scale assembly and effective additive presentation.^24^, ^27^, ^28^ The current results confirm that in conjunction to the employed supramolecular additive, also the polymer backbone influenced nano‐scale assembly of both UPy and BU‐fibers, which in turn can affect additive effectiveness.

## 4 CONCLUSIONS

This study demonstrated the importance of both the polymer backbone and the additive in supramolecular elastomeric biomaterials. Additives in UPy‐based materials appeared to be less intrusive on the nano‐scale assembly observed at surface, with low levels of aggregates observed. Depending on the polymer backbone assembled nano‐fibers were either elongated or truncated. UPy‐Catechol modification appeared to be more effective for cell adhesion in PriplastdiUPy than in PCLdiUPy, and vice versa for UPy‐cRGD modifications. Within the BU‐system additive addition was found to be highly intrusive on the nano‐scale surface assembly of fibers. BU‐Catechol modification led to hydrophobic platelet formation on the surface, which was detrimental for cell adhesion with serum present in culture conditions, but highly effective for long‐term adhesion without serum. BU‐cRGD addition induced fibril aggregates on the surface in PCL‐BU; however, when incorporated in PC‐BU elongated fibers with local alignment appeared on the surface. cRGD modification was found to be most effective in PCL‐BU over PC‐BU and the UPy‐system polymers. This research highlighted the importance of reevaluating the effectiveness of cell adhesive additives when translating from one polymer backbone to another, and from one supramolecular system to another, to suit the biomaterial application. Additive transposition might not always be as evident as desired, and should be (re)evaluated per cell type and polymer‐additive combination.

## Supporting information


**Appendix** **S1: Supporting information**
