# Supplementary material for: Effectiveness of cell adhesive additives in different supramolecular polymers
Source: J Polym Sci (2020). 2021 Mar 29;59(12):1253–66. doi: 10.1002/pol.20210073 (PMC8252730; doi:10.1002/pol.20210073)
Supplement: Supplementary file 1 — Appendix S1: Supporting information [file POLA-59-1253-s001.pdf]

Supporting Information: Effectiveness of cell adhesive additives in different supramolecular polymers

Ronald C. van Gaal[1,2], Bastiaan D. Ippel[1,2], Sergio Spaans[1,2], Muhabbat I. Komil[2,3], Patricia Y.W. Dankers[2,3]

[1]Laboratory for Cell and Tissue Engineering, Department of Biomedical Engineering, [2]Institute for Complex Molecular Systems, [3]Laboratory of Chemical Biology, Department of Biomedical Engineering, Eindhoven University of Technology, PO Box 513, 5600 MB, Eindhoven, The Netherlands

Correspondence to: Patricia Y.W. Dankers (E-mail: p.y.w.dankers@tue.nl)

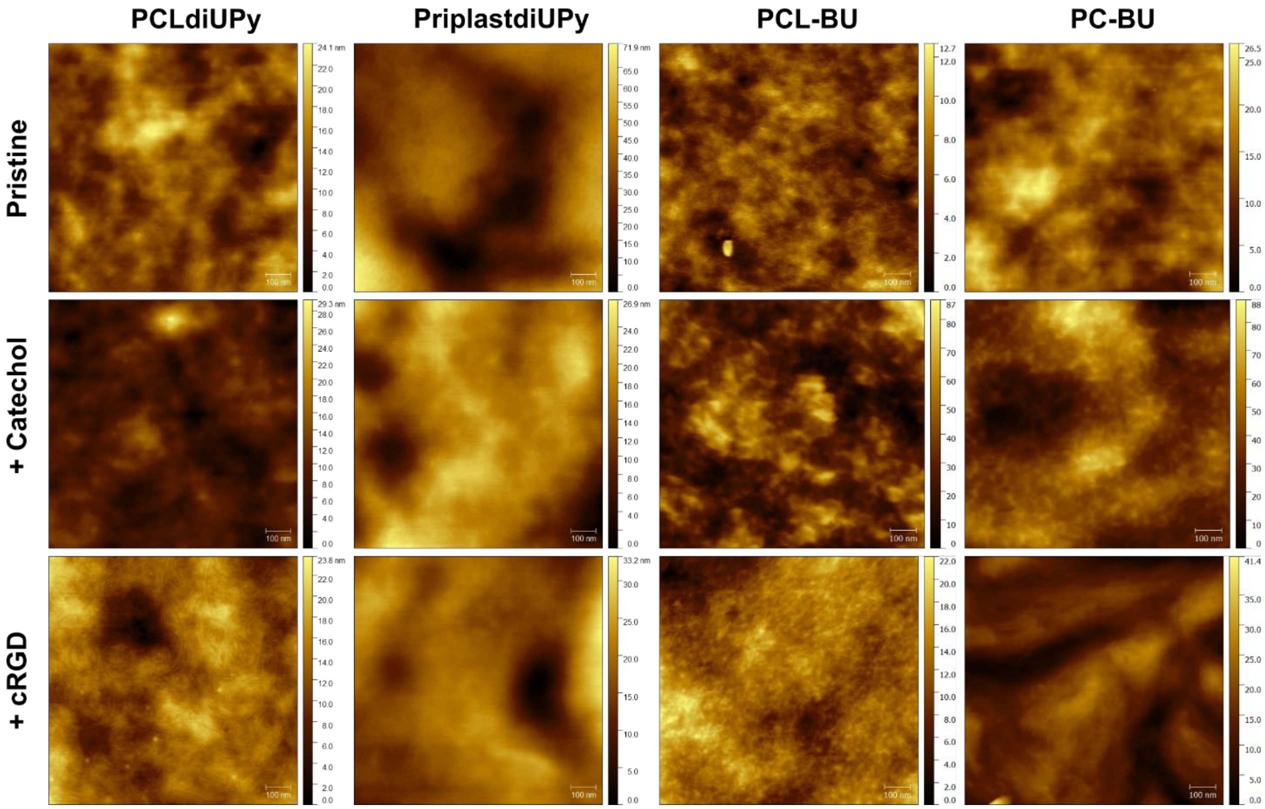

FIGURE S1 Influence of backbone polymer and additive on surface height. AFM phase micrographs of solution cast films of polymer and additive combinations. Selected polymers from left to right, PCLdiUPy, PriplastdiUPy, PCL-BU, and PC-BU. Additive functionalization from top to bottom, no additive (pristine), 5 mol% UPy-Catechol or BU-Catechol applied in corresponding supramolecular base polymer (Catechol), and 5 mol% UPy-cRGD or BU-cRGD applied in corresponding supramolecular base polymer (cRGD). Scale bar is 100 nm.

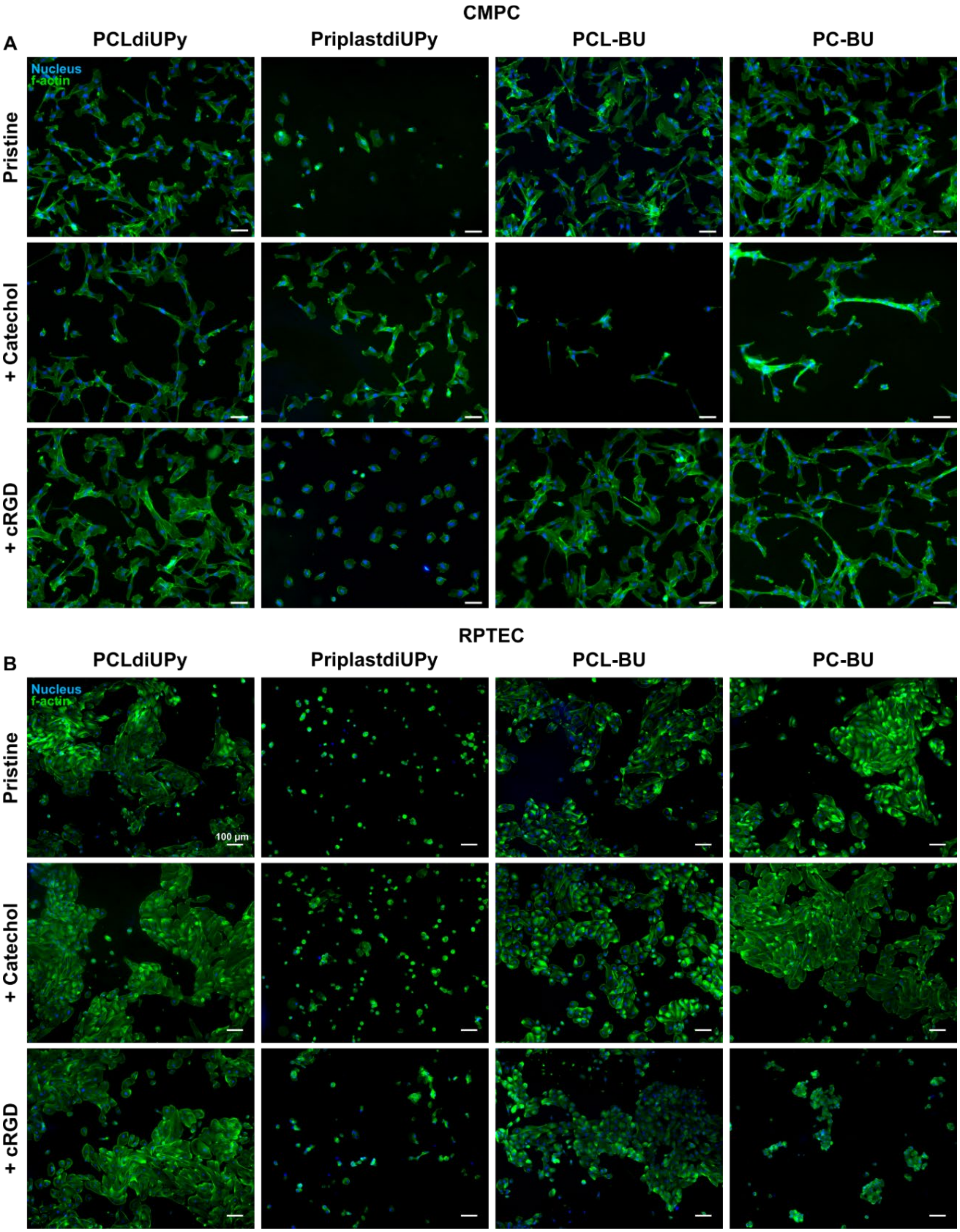

FIGURE S2 CMPC and RPTEC adhesion to supramolecular materials. **A** CMPCs or **B** RPTECs cultured for 24h on biomaterials, stained for f-actin (green) and the nucleus (blue), scale bars represent 100 µm. Selected polymers from left to right, PCLdiUPy, PriplastdiUPy, PCL-BU, and PC-BU. Additive functionalization from top to bottom, no additive (Pristine), 5 mol% UPy-Catechol or BU-Catechol applied in corresponding supramolecular base polymer (Catechol), and 5 mol% UPy-cRGD or BU-cRGD applied in corresponding supramolecular base polymer (cRGD).

WILEY ONLINE LIBRARY

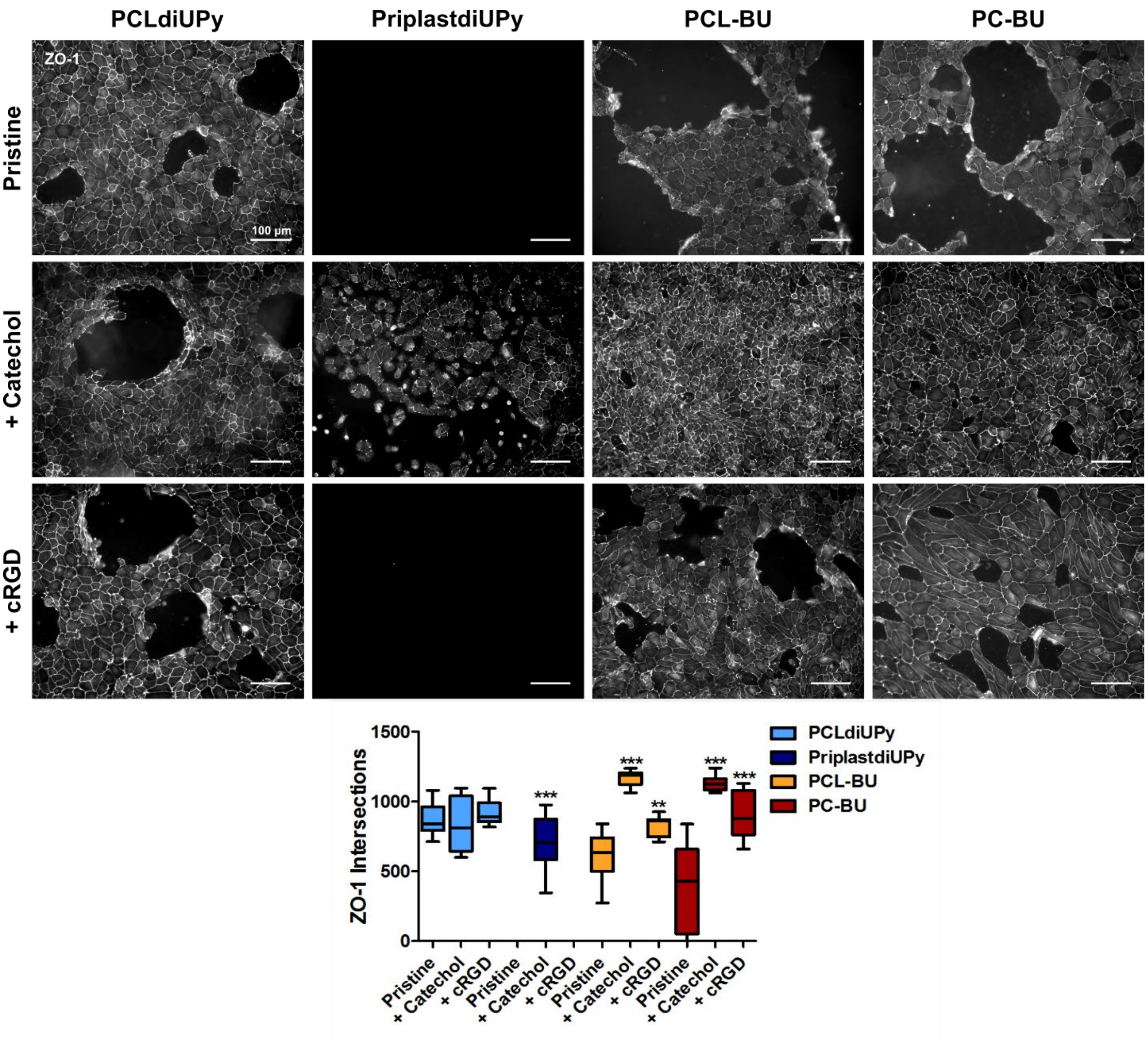

FIGURE S3 Long-term monolayer formation on supramolecular materials. RPTEC cultured on biomaterials for 3 weeks and stained for Zona Occludens-1 (ZO-1), scale bar is 100 µm. Selected polymers from left to right, PCLdiUPy, PriplastdiUPy, PCL-BU, and PC-BU. Additive functionalization from top to bottom, no additive (Pristine), 5 mol% UPy-Catechol or BU-Catechol applied in corresponding supramolecular base polymer (Catechol), and 5 mol% UPy-cRGD or BU-cRGD applied in corresponding supramolecular base polymer (cRGD). Bottom graph depicts quantification of ZO-1 per image, data derived from three replicates, mean ± standard error of the mean presented. $^{**}p \leq 0.01$, $^{***}p \leq 0.001$ compared to pristine.

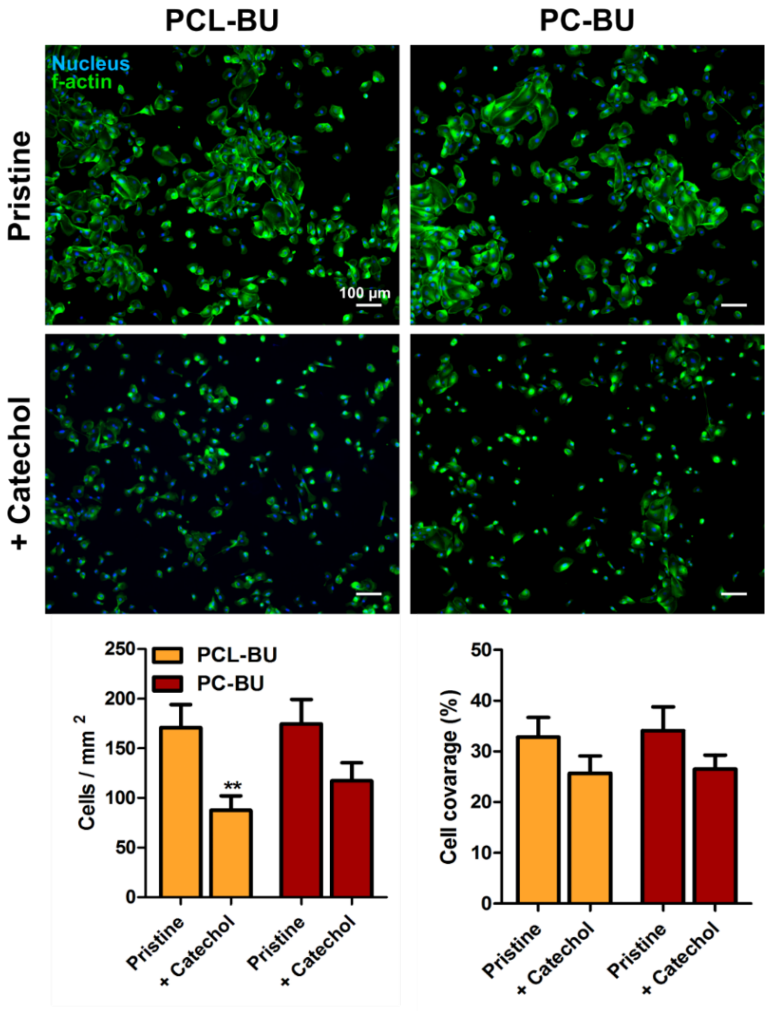

FIGURE S4 Influence of serum on RPTEC adhesion to pristine and catechol modified materials. RPTECs cultured for 24h on biomaterials, stained for f-actin (green) and the nucleus (blue), scale bar is 100 µm. Selected polymers from left to right, PCL-BU, and PC-BU. Additive functionalization from top to bottom, no additive (Pristine), and 5 mol% UPy-Catechol or BU-Catechol applied in corresponding supramolecular base polymer (Catechol). Bottom graphs depict quantification of cells/mm^2 (left) and cell coverage over the biomaterial surface (right) from fluorescent images. Data of three replicates, mean ± standard error of the mean. **p≤0.01 compared to pristine.

WILEY ONLINE LIBRARY

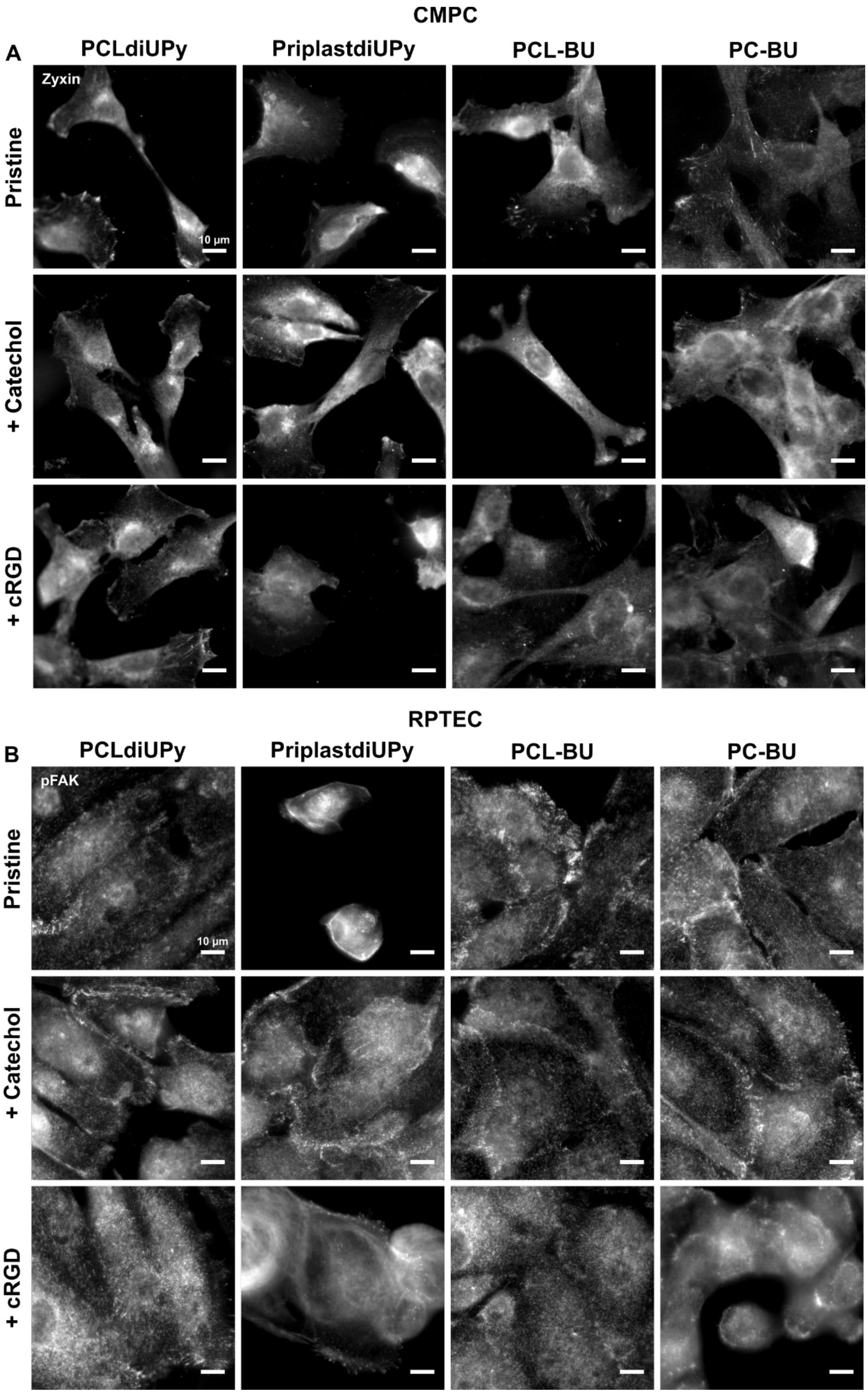
CMPC
A
PCLdiUPy
PriplastdiUPy
PCL-BU
PC-BU
Zyxin
Pristine
10 µm
+ Catechol
+ cRGD
RPTEC
B
PCLdiUPy
PriplastdiUPy
PCL-BU
PC-BU
pFAK
Pristine
10 µm
+ Catechol
+ cRGD

FIGURE S5 Focal adhesions behavior of CMPC and RPTEC on supramolecular biomaterials. Fluorescence microscopy images of **A** CMPCs or **B** RPTECs cultured for 24h on PCLdiUPy (left column), PriplastdiUPy (center left column), PCL-BU (center right), or PC-BU (right column) films. Additive functionalization from top to bottom, no additive (pristine), 5 mol% UPy-Catechol or BU-Catechol applied in corresponding supramolecular base polymer (Catechol), and 5 mol% UPy-cRGD or BU-cRGD applied in corresponding supramolecular base polymer (cRGD). Cells stained for zyxin, scale bars are 10 µm, *n=3*.

WILEY ONLINE LIBRARY
